# Prevalence of rheumatic diseases in Raramuri people in Chihuahua, Mexico: a community-based study

**DOI:** 10.1007/s10067-016-3225-x

**Published:** 2016-03-08

**Authors:** Danyella Del Río Nájera, Natalia Santana, Ingris Peláez-Ballestas, Susana A. González-Chávez, Celia M. Quiñonez-Flores, César Pacheco-Tena

**Affiliations:** 1 Facultad de Medicina, Universidad Autónoma de Chihuahua, Circuito No. 1, Nuevo Campus Universitario, C.P. 31240 Chihuahua, CHIH Mexico; 2 Instituto Mexicano del Seguro Social, Hospital de Especialidades Morelos, Chihuahua, Mexico; 3 Rheumatology Department, General Hospital of México “Eduardo Liceaga”, Mexico City, Mexico

**Keywords:** COPCORD, Indigenous people, Mexico, Musculoskeletal pain, Rheumatic diseases

## Abstract

This study aimed to determine the prevalence of musculoskeletal (MSK) pain and rheumatic diseases in the Raramuri population (also known as Tarahumaras) who are an indigenous group in the northern state of Chihuahua in Mexico. We used the Community-Oriented Program for Control of Rheumatic Diseases (COPCORD) methodology. An analytical cross-sectional study was conducted including indigenous Raramuri aged ≥18 years from communities settled in Chihuahua City. Subjects with positive MSK pain were evaluated by primary care physicians and rheumatologists. Demographic and occupational factors such as gender and job type associated with rheumatic disease were investigated. A total of 380 indigenous Raramuri (mean age 33.6 ± 13.1 years; 37.9 % male) were interviewed. Seventy-six individuals (20 %) reported MSK pain in the last 7 days. Pain intensity was reported as “severe” and “the most severe” in 30 % of the cases. Fifty-six individuals (14.7 %) reported pain in the past and 86 (22.6 %) had either past or current pain. The prevalence of rheumatic diseases was 10.5 %. Diagnosed diseases were osteoarthritis (6.6 %), low back pain (1.6 %), spondyloarthritis (0.8 %), rheumatoid arthritis (0.5 %), non-specific arthritis (0.5 %), rheumatic regional pain syndromes (0.3 %), and fibromyalgia (0.3 %). Rheumatic disease was associated with the following variables: age (odds ratio (OR) 1.04, 95 % confidence interval (CI) 1.02–1.08; *p* = 0.006), family history of rheumatic symptoms (OR 6.9; 95 % CI 2.6–18.7; *p* < 0.001), and Health Assessment Questionnaire-Disability Index (OR 28.9; 95 % CI 2.8–289.7; *p* < 0.001). A high prevalence of non-traumatic MSK pain suggests the need for a rheumatic disease prevention program in the Raramuri people in Chihuahua, Mexico.

## Introduction

Rheumatic diseases represent a global problem for both patients and healthcare providers. Developing countries are particularly vulnerable because of insufficient and delayed diagnosis of these diseases [1, 2]. Pain in the musculoskeletal (MSK) system is characteristic of rheumatic diseases and, additionally, may lead to disability. Individuals suffering from rheumatic disorders have a significant reduction in both their quality of life and life expectancy [3].

The Community-Oriented Program for Control of Rheumatic Diseases (COPCORD) was founded by the World Health Organization and International League of Associations for Rheumatology. This program emerged as a strategy to define the epidemiologic landscape of rheumatic diseases in developing countries. COPCORD provides a screening tool for rheumatic diseases for low-cost community studies [2, 4].

The COPCORD methodology has already been used to assess the prevalence of MSK disorders and rheumatic diseases in Latin American countries [5–9]. In Mexico, this methodology has been applied in a number of distinct geographic regions [3, 10–13]. Previous studies focused mostly on Mexican mestizo populations. COPCORD was also applied in more susceptible populations including indigenous groups [7, 14]. A culture-sensitive adaptation and validation of this methodology was performed in Latin American indigenous populations. It included Warao, Kariña, and Chaima (Venezuela); Mixteco, Maya-Yucateco, and Raramuri (Mexico); and Qom (Argentina) groups [15].

Mexico has a multicultural environment that includes many indigenous groups [16, 17]. The Raramuri people (also known as Tarahumaras) are a minority indigenous people living in the northern region of Mexico. The main location of this group is in the states of Chihuahua, Durango, and Sonora. The Raramuri people living in Chihuahua comprise a group of 158,527 people, which is almost 5 % of the state’s total population [17].

The Raramuri people are considered as a vulnerable group [18]. Their main environment is the “Sierra Tarahumara,” which is a rural region composed of mountains, canyons, and thick forests. The terrain is vast and inaccessible, resulting in poor Raramuri health status given their very limited or absent access to healthcare facilities [18–20]. There is a high rate of infant mortality because of infant malnutrition and susceptibility to various infectious diseases [20]. Although poor health conditions have been recognized, to our knowledge, the prevalence of chronic illnesses, including MSK disorders and rheumatic diseases, has not been reported in the Raramuri indigenous population. However, it has been found that 3.7 % of this population in Chihuahua State has some kind of physical impairment [17] which could be associated with MSK disorders. Detailed information regarding the prevalence of rheumatic diseases could help to create specific prevention programs and also improve healthcare provision in this group. Thus, the aim of this study was to estimate the prevalence of MSK regional pain syndromes (neck, shoulder, etc.) and defined rheumatic diseases in Raramuri individuals over 18 years of age living in Chihuahua City, as well as to determine the factors associated with the presence of these rheumatic diseases.

## Materials and methods

### Study design

A cross-sectional community-based study was conducted using the COPCORD methodology. The screening tool applied was the COPCORD questionnaire which has been adapted and validated for the Raramuri people [15]. The subjects included in the study were aged 18 years or older and belonged to the Raramuri community. They were invited to take part in the study and voluntarily gave consent to participate in the survey.

### Screening tools

The COPCORD questionnaire [15] included items related to (1) sociodemographic factors; (2) comorbidities; (3) symptoms: pain, swelling, and stiffness; (4) pain characteristics: period of time (last 7 days or historic pain), intensity [five options in the Likert scale ranging from none to more severe pain], and location; and (5) treatment: use of conventional and non-conventional medicine. Moreover, information about disability and work biomechanical loading was collected from the subjects affected by MSK pain using the Health Assessment Questionnaire-Disability Index (HAQ-DI) [21] and the mechanical stress (dynamic and static) questionnaire [22], respectively. The mechanical stress questionnaire considers the kind of work that individuals have done throughout their life and identifies the types of positions and movements performed during work. Both questionnaires were applied by trained personnel.

### Study population

Chihuahua is one of the 32 federal entities in Mexico. Its territory covers more than 250,000 km^2^ and is the largest in the country. Chihuahua is divided into 67 municipalities, with Chihuahua City as its capital. Chihuahua City has 819,543 habitants [23], 13,209 (1.6 %) of whom belong to the Raramuri indigenous group. Of this group, 8948 people are aged 15 years or older. Nearly half of the Raramuri population (48.6 %) in Chihuahua City speaks the native language Raramuri whereas 88.6 % speak Spanish. Bilingualism is reported by 37.2 % of the Raramuri indigenous population. However, 51.4 % of this indigenous group are monolingual in Spanish, only 0.4 % are monolingual in Raramuri, and the remaining 11.0 % speak Raramuri but do not specify bilingualism [17]. Although these figures are official, a new (unpublished) survey done by the state government in November 2014 showed a further advance on the preference toward Spanish language in the Raramuri at least in the cities. This survey included 2601 Raramuri residing in Chihuahua City, and 68 % of them are bilingual. It also shows a growing proportion of Spanish-only speakers.

In regard to health services, a total of 9283 subjects are covered by a government-based healthcare system [18]. In their indigenous lands, the Raramuri earn their living with agriculture and goat shepherding; but it is fair to mention that their soils are far from adequate for such tasks. They lack technological aids and are impoverished as a rule; therefore, they rely on nature’s whim and primitive tools to actually harvest something. Children are frequently part of the workforce and education is not a priority. Several famines have struck Raramuri historically and malnutrition is a common denominator.

While the Raramuri mostly remain as a rural ethnicity, a clear trend toward migration and westernization is evident within the last decades. Climatic change and several sociological issues (such as drug trafficking and its violence within the region) have progressively expelled Raramuri from their original settings into villages and cities. As they come in within the mestizo social structures, they are frequently hired to carry low-payment jobs with very high physical demand (such as construction in the lowest-rank laborer). Language and cultural barriers preclude them as a rule from high education; nevertheless, their situation within Mexican mestizo societies is more convenient; as an example, at their indigenous settings, they live in great isolation either in caves or in the best of cases in primitive wood cabins lacking fundamental commodities as running water or stool disposal. At the city-based settlements, they live in small brick houses where commodities are seldom available. This precarious improvement and the progressive generational blending with the Mexican mestizo culture allow them to gain further educational and job opportunities, and likely, these explain the migratory trend.

For this study, we had to select the urban Raramuri population rather than the native population in the forest based on the feasibility of reaching a large indigenous population in a brief period. The forest settlements include very few individuals who are dispersed over a vast, inaccessible terrain. The Chihuahua government, through the Department of Coordination of the Tarahumara (Spanish: Coordinadora Estatal de la Tarahumara (CET)), has a register of 27 permanent Raramuri settlements in Chihuahua City in which 1291 individuals aged 18 years or older are established. In the settlements, about 942 Raramuris have studied or are studying in elementary school, 298 in middle school, and 105 in high school.

The sample size was calculated using the formula for a finite population (1291). The confidence level was calculated at 95 % with a margin of 5 %. The estimated value was 297. We restricted our interviews to a proportion of the settlements (16 out of 27) for convenience. Thus, we included those settlements with access granted by the settlement governor, and we targeted settlements with a higher population and with ease of access within the city. The sampled settlements included over 85 % of the Raramuri population in Chihuahua City.

### Survey

The study was conducted between May 2013 and January 2014. Six interviewers, who were social work college students, applied the questionnaire with the assistance of local Raramuri translators. They received specific training in the use of the COPCORD questionnaire and also in the overall research methodology. The interviewers visited each house in the selected Raramuri settlements and invited inhabitants older than 18 years to participate in the survey. If they accepted, COPCORD questionnaire was applied. The data obtained in the interviews were collected and stored in a database for subsequent analysis.

Cases were considered COPCORD-positive if they had pain/stiffness/swelling in the previous 7 days and/or previously. Primary care physicians then assessed COPCORD-positive subjects to identify potential rheumatic disease in them. If there was suspicion of a rheumatic disease, the patient was clinically assessed by a board-certified rheumatologist to establish a specific diagnosis using established classification criteria [24–31].

### Statistical analysis

A descriptive analysis with measures of central tendency and dispersion for continuous variables was performed. Absolute and relative frequencies for categorical variables were determined. An inferential analysis was initially performed by uni- and bi-directional analyses of variance for continuous variables. For categorical variables, the chi-square test was used and considered statistically significant if *p* ≤ 0.05*.*

A logistic regression analysis was performed with forward steps, with diagnosis of a rheumatic disease as the outcome variable. Odds ratios (ORs) and 95 % confidence intervals (CIs) were determined. Independent variables included those with clinical relevance and/or statistical significance (*p* ≤ 0.05): sociodemographic variables, currently working, work involving loads, repetitive work, pain in the last 7 days, pain intensity, some kind of treatment received, and functional disability measured by HAQ-DI; the score had a cutoff of 0.8 or above to define physical impairment [13]. This analysis aimed to identify those variables associated with the presence of rheumatic disease, which could be used as a proxy predictor to derived individuals from primary care to rheumatology consultation. The statistical analyses were performed using Stata SE version 11.0 for Mac.

### Ethical aspects

This study was approved by the local ethical committee of the Autonomous University of Chihuahua. A complete explanation of the objective, strategy, and data confidentiality of the study was given to the participants. Those interviewed gave their consent to participate in the survey verbally; since overall consent was granted by their governors, no written consent was deemed necessary according to the opinion of the governors. The state government responsible for Raramuri affairs granted an appointment with the Raramuri governors of all the settlements, and the purpose and structure of the COPCORD strategy were presented in both Spanish and Raramuri languages with the aid of translators. Overall, the response of the governors was favorable; however, the time permitted for accessing the settlements was limited to a few weeks. In most cases, returning for applying the COPCORD questionnaire to expand the sample or a second interview for more details was not an option.

## Results

A total of 380 subjects (29.4 %) among 1291 people aged ≥18 years living in Raramuri settlements were included in this study. Table 1 describes the sociodemographic characteristics of the population. The study group was composed of 236 (62.1 %) women and 144 (37.9 %) men, with a mean age of 33.6 years (SD 13.1 years). Only 16.1 % of the indigenous were born in the Chihuahua municipality. The rest of them had migrated from the Sierra Tarahumara (53.7 %) or from other municipalities in the state near the Sierra (30.3 %). More than half (52.6 %) of the studied population was employed. Over 50 % of the interviewed subjects reported having worked in activities involving frequent exposure to static or dynamic biomechanical stress (Table 2).

**Table 1:** Demographic and socioeconomic characteristics of the study population (*n* = 380)

Variables	Result^a^
Gender	
Women	236 (62.1)
Age, mean (SD), years	33.6 (13.1)
Marital status	
Married	273 (71.8)
Single	107 (28.1)
Occupation (*n* = 357)^b^	
Housekeeping	133 (37.3)
Construction	69 (19.3)
Domestic service	58 (16.2)
Retired/student	18 (5.0)
Laborer/industry	17 (4.8)
Seller/employee	15 (4.2)
Farmer	11 (3.1)
Others	36 (10.1)
Employed	200 (52.6)
Unemployed	180 (47.4)
Cause (*n* = 180)	
Unable to be hired	102 (56.7)
Home and children care	32 (17.8)
Health problems	27 (15.0)
Job problems	2 (1.1)
Other problems	7 (3.9)
Aged	4 (2.2)
Others	6 (3.3)

*SD* standard deviation

^a^Unless otherwise specified, values are depicted as *n* (%)

^b^Only 357 of 380 individuals agreed to answer the question (response rate 93.9 %)

**Table 2:** Work biomechanical stress patterns (*n* = 380)

Variable	*n* (%)
Dynamic mechanical stress	
Shaking hands	289 (76.1)
Pushing an object >20 kg	199 (52.4)
Handling load >20 kg	206 (54.2)
Frequently going up or down strain	146 (38.4)
Walking for over 30 min	282 (74.2)
Frequently standing and sitting	219 (57.6)
Static mechanical stress	
Standing for over 30 min	287 (75.5)
Bending down for over 30 min	227 (59.7)

The most reported comorbidity was alcoholism 123 (32.4 %), followed by depression 80 (21.0 %), smoking 72 (18.9 %), anxiety 64 (16.8 %), gastritis 60 (15.8 %), obesity 56 (14.7 %), systemic arterial hypertension (SAHT) 45 (11.8 %), heart disease 35 (9.2 %), vascular disease 35 (9.2 %), type 2 diabetes 29 (7.7 %), hyperlipidemia 19 (5.0 %), drug abuse 11 (2.9 %), and others (infections, asthma, and history of trauma) 25 (6.6 %). In our sample, 16.8 % had a family member with a rheumatic disease.

The characteristics of subjects who reported current or past MSK pain are described in Table 3. MSK pain in the last 7 days was reported in 76 (20.0 %; 95 % CI 16.3–23.9) individuals, of whom 54 (71.1 %) reported that their pain was not related to a previous trauma*.* Past pain was present in 56 subjects (14.7 %; 95 % CI 11.6–18.8), of whom 37 (66.1 %) reported non-trauma history. Eighty-six (22.6 %) individuals were classified as COPCORD-positive. When asked whether they had seen a doctor and received a diagnosis, 48/55 (87.3 %) answered affirmatively, of whom 45 (81.8 %) reported medical care from a doctor, while only three (5.5 %) reported using traditional or alternative medical treatment. Forty-three (78.2 %) individuals received some type of medication.

**Table 3:** Characteristics of subjects with reports of current and past MSK pain (*n* = 380)

Variables	*n* (%)
MSK pain in the last 7 days	76 (20.0)
Trauma-related	22 (28.9)
Non-trauma-related	54 (71.1)
Pain intensity (VAS), *n* = 76	
None	10 (13.2)
Some pain	20 (26.3)
Mild pain	16 (21.1)
Severe	17 (22.4)
The most severe pain	13 (17.1)
Past history of MSK pain	56 (14.7)
Trauma-related	19 (33.9)
Non-trauma-related	37 (66.1)
Pain intensity, *n* = 56	
None	4 (7.1)
Some pain	8 (14.3)
Mild pain	15 (26.8)
Severe	14 (25.0)
The most severe pain	14 (25.0)
MSK pain in the last 7 days and past history of pain	46 (12.1)
Familiar history of rheumatic symptoms	64 (16.8)
Medical care^a^	48 (87.3)
Biomedical or non-homeopathic care	45 (81.8)
Traditional-alternative medical care^b^	3 (5.5)
Treatment^a^	43 (78.2)
NSAID	23 (53.4)
Alternative	9 (20.9)
Others^c^	24 (55.8)

*MSK* musculoskeletal, *NSAID* non-steroidal anti-inflammatory drugs, *VAS* visual analog scale

^a^Only 55 of 86 individuals (response rate 63.9 %) agreed to answer the question

^b^Spiritualists, bonesetters, herbalists, chiropractors

^c^Ointments and non-specified treatment

**Fig. 1:**
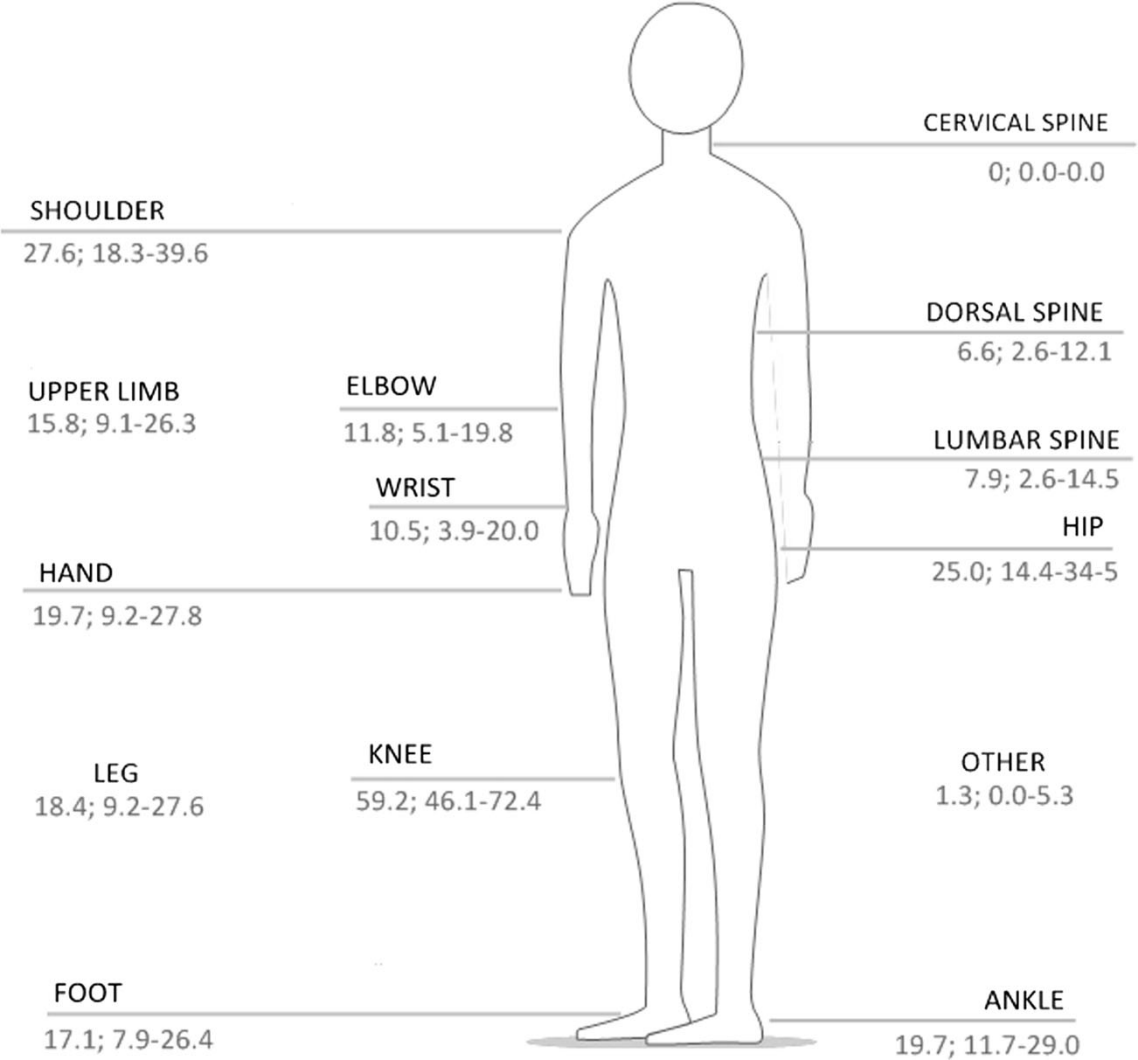
Body regions affected by pain in the last 7 days

MSK pain in the last 7 days was reported as monoarticular (31.6 %), oligoarticular (36.8 %), or polyarticular (31.6 %). Body regions affected by pain in the last 7 days are represented in Fig. 1. Additionally, the frequencies of pain in most affected body regions both within the last 7 days and in the past, respectively, were in the knees (59.2 and 50.0 %), shoulders (27.6 and 28.6 %), hip (25.0 and 26.8 %), hand (19.7 and 19.6 %), ankle (19.7 and 12.5 %), legs (18.4 and 16.1 %), and feet (17.1 and 17.9 %).

The functional impact of MSK symptoms measured by HAQ-DI was evaluated in the 86 COPCORD-positive subjects. In total, 38 (44.2 %) subjects reported that they had some physical limitation at the time of interview. Only 2 (2.3 %) subjects reported to be physically impaired in the past. The COPCORD-positive subjects reported their discomfort as “none,” “some pain,” “mild pain,” “severe pain,” and “the most severe pain” in 13 (15.1 %), 23 (26.7 %), 18 (20.9 %), 19 (22.1 %), and 13 (15.1 %) of the cases, respectively. The level of coping with discomfort was answered by 80 of the COPCORD-positive subjects, with none, some, good, and excellent coping in 8 (10.0 %), 24 (30.0 %), 33 (41.3 %), and 15 (18.8 %), respectively. The median HAQ-DI score was 0.1 and the inter-quartile range (IQR) was 0.0–0.325.

The prevalence of rheumatic diseases was 10.5 % (95 % CI 7.2–13.5). The most frequent diagnosis was osteoarthritis (OA) in 6.6 % (95 % CI 4.2–8.9) (Table 4). Univariate comparisons of sociodemographic and clinical factors of individuals with rheumatic disease and controls (COPCORD-negative) are shown in Table 5. The variables age, family history of rheumatic symptoms, HAQ-DI, and the self-reported comorbidities (obesity, systemic arterial hypertension, and depression) were significantly different (*p* ≤ 0.05) between the groups.

**Table 4:** Prevalence of rheumatic diseases and MSK disorders (*n* = 380)

Diagnostic	Cases	Prevalence (%)	95 % CI
COPCORD-negative	315	82.9	79.1–86.3
Rheumatic disease	40	10.5	7.2–13.5
Osteoarthritis	25	6.6	4.2–8.9
Low back pain	6	1.6	0.5–2.6
Ankylosing spondylitis	3	0.8	0.0–1.8
Rheumatoid arthritis	2	0.5	0.0–1.3
Non-specific arthritis	2	0.5	0.0–1.6
RRPS	1	0.3	0.0–0.8
Fibromyalgia	1	0.3	0.0–0.8
MSK disorders	25	6.6	4.3–8.7
Associated with neurological disorders	6	1.6	0.5–3.2
Associated with orthopedic disorders	16	4.2	2.4–6.6
Others	3	0.8	0.0–1.8

*CI* confidence interval, *COPCORD* Community-Oriented Program for Control of Rheumatic Diseases, *RRPS* rheumatic regional pain syndrome, *MSK* musculoskeletal

**Table 5:** Comparison of individuals with rheumatic disease and controls (COPCORD-negative)

Variables	Diagnostic of a rheumatic disease^c^, *n* = 40	COPCORD-negative (controls)^c^, *n* = 315	*p* value
Gender^a^	23 (57.5)	185 (63.1)	0.4
Age, mean (SD)^b^	42.1 (13.2)	32.2 (12.2)	<0.01
Familiar history of rheumatic symptoms	17 (42.5)	24 (8.1)	<0.01
Current work	20 (50)	144 (49.1)	0.9
Dynamic mechanical stress			
Shaking hands	30 (75)	223 (76.1)	0.8
Pushing an object >20 kg	26 (65)	153 (52.2)	0.1
Handling load >20 kg	26 (65)	156 (53.2)	0.1
Frequently going up or down strain	17 (42.5)	107 (36.5)	0.4
Walking for over 30 min	30 (75)	214 (73.0)	0.7
Frequently standing and sitting	23 (57.5)	164 (55.9)	0.8
Static mechanical stress			
Standing for over 30 min	33 (82.5)	217 (74.0)	0.2
Bending down for over 30 min	28 (70)	166 (56.6)	0.1
HAQ-DI	0.23 (0.27)	0.01 (0.05)	<0.01
Self-reported comorbidities			
Alcoholism	17 (42.5)	89 (30.3)	0.1
Depression	17 (42.5)	45 (15.3)	<0.01
Smoking	11 (27.5)	51 (17.4)	0.1
Anxiety	8 (20)	42 (14.3)	0.3
Obesity	11 (27.5)	34 (11.6)	0.006
SAHT	12 (30)	23 (7.8)	<0.01
Type 2 diabetes mellitus	5 (12.5)	15 (5.1)	0.06

*COPCORD* Community-Oriented Program for Control of Rheumatic Diseases, *SD* standard deviation, *HAQ-DI* Health Assessment Questionnaire-Disability Index, *SAHT* systemic arterial hypertension

^a^Chi-square test was used for dichotomic or nominal variables

^b^ *t* test was used for numerical variables

^c^Unless otherwise specified, values are depicted as *n* (%)

To determine the variables associated with the presence of rheumatic disease, a multiple logistic regression analysis, starting with an unsaturated design and adding variables one by one, was performed. The dependent variable was a rheumatic disease diagnosed by a rheumatologist. The independent variables selected were those that resulted significant in the bivariate analysis or variables that have biological plausibility. There was a significant association between the presence of rheumatic disease and the following variables: age (OR 1.04; 95 % CI 1.02, 1.08; *p* = 0.006), family history of rheumatic symptom (OR 6.9; 95 % CI 2.6–18.7; *p* < 0.001), and HAQ-DI (OR 28.9; 95 % CI 2.8–289.7; *p* < 0.001). According to our model, these variables account for 40 % of the likelihood of a rheumatic disease being present *(p* = 0.004).

## Discussion

Deficient health care is a part of the burden in vulnerable populations. MSK pain and rheumatic diseases are complicated syndromes that depend on expert medical practitioners for early diagnosis and specific treatment within the therapeutic window at early stages of the diseases. Several aspects such as diagnosis delay for specific diseases in vulnerable groups cannot be extrapolated from the reference population around it, but should be assessed specifically; frequently, ethnical and cultural barriers create different environments even in the same area.

The COPCORD program has been used previously in indigenous populations such as the Kaqchiquels in Guatemala [7] or Australian aborigines [14]. Our study in the Raramuri indigenous group in Chihuahua, Mexico, showed a lower prevalence of MSK pain (20 % recently and 14.7 % in the past) compared to both the Guatemalan Kaqchiquels (60.9 %) and the Australian aborigines (33 %). The differences could be explained by genetic factors [32] and also by work and cultural and environmental factors [16, 17].

Although the Raramuri group was found to have a lower prevalence of MSK compared with other ethnic populations, nearly three quarters (71.1 %) of the population had experienced MSK pain unrelated to trauma in the previous 7 days. These cases can be considered as potentially having a rheumatic disease. In addition, physical limitation was reported in near half of these patients (46.5 %). This number of COPCORD-positive cases and the prevalence of disability should be of concern to healthcare authorities by the potential burden on both society and healthcare systems.

The most common location of MSK pain in the last 7 days and historically was in the knees, followed by the shoulders, hip, hand, ankle, legs, and feet. This pattern resembled that reported for Guatemalan subjects, but in Australian aborigines, the spine was the most common site. Women predominated in our sample (62.1 %), similar to the Guatemalan study. The female predominance in the COPCORD-positive group (58.1 %) likely influenced the pain topography; most Raramuri women depend on walking for transportation, and for prolonged stages in their life, they carry their offspring or younger siblings in their *rebozos* (part of a Raramuri woman’s clothing used to load a child on her back). Therefore, strain is placed on the knees and shoulders. For males, construction was the commonest job and mainly involved laboring, which would consequently affect the shoulders and hands.

The prevalence of rheumatic diseases in the Raramuri population was 10.5 %, which is higher than that reported in the Guatemalan population (8.23 %), but lower than that reported in Australian aborigines (33 %). No specific explanation for these differences is found in the methodology, so probably genetic and environmental factors are accountable. OA was the most prevalent rheumatic disease in the Raramuri population (6.6 %) and was similar to the prevalence in the mestizo population in Mexico [3] and in many other countries worldwide [6, 7, 9, 33–35]. OA is the most common form of arthritis and is strongly associated with aging [36] and with high mechanical demand [37]. In the Guatemalan population, OA was also the commonest condition while in Australian aborigines it was the second most common after rheumatic regional pain syndromes (RRPS). In contrast, RRPS was the least common rheumatic disease in Raramuris (0.3 %). RA was diagnosed in 0.5 % of our sample and in 0.85 % of the Guatemalans, but no figure was given for Australian aborigines. OA and RA prevalence are very likely underestimations given the mean age of the population sampled.

The prevalence of low back pain was higher in the Raramuri population (1.6 %) compared with the Guatemalan population (0.5 %) and was lower than that in Australian aborigines (4.3 %). The high prevalence in aborigines may be related to the high prevalence of obesity, which confers a mechanical load on most body joints, thereby contributing to the development of low back pain. Obesity was observed in 14.7 % of Raramuris and, while not ideal, was lower than the national average of 32.4 % (Mexico is ranked second worldwide in obesity for both general and pediatric populations) [38]. Obesity was more prevalent in Raramuris than in the Guatemalan indigenous population (10 %), but far lower than in Australian aborigines (58 %).

The most frequent comorbid condition in Raramuris was alcoholism (32.4 %), which was lower than that reported in Australian aborigines, followed by depression, anxiety, and tobacco smoking. These conditions are frequently interrelated and likely reflect the social adversity experienced by this population, with a high degree of physical activity and probably restricted access to food. Diabetes mellitus was observed in 7.7 % of Raramuris, similar to the Guatemalan population (6 %) and lower than in Australian aborigines (12.5 %). Arterial hypertension was lower in Raramuris (11.8 %) versus 20 and 24 % in Guatemalan and Australian populations, respectively. It should be noted that the mean age of the Raramuri population in this study was very similar to those of both the Guatemalan and Australian aborigine populations (35 years in both); therefore, the comparison of the prevalence of comorbidities is valid. The prevalence of diabetes and arterial hypertension increases with age and may be underestimated in these populations with a low mean age [39].

In the regression analysis, several parameters correlated with the presence of rheumatic diseases. As expected, an older age, a family history of rheumatic symptoms, and higher HAQ-DI scores conferred an increased risk to rheumatic disease. Diabetes and arterial hypertension also were higher in patients with rheumatic diseases; however, since the patients with rheumatic diseases were older, this difference is attributable at least partially to the natural history of diabetes and hypertension. It should be noted, however, that inflammatory rheumatic diseases do indeed increase the cardiovascular risk. Obesity confers risk for osteoarthritis and other mechanically induced conditions, and also, the reduced mobility of the patients with rheumatic diseases predisposes to obesity.

Depression was also more frequent among patients with rheumatic diseases; in our opinion, this association can be interpreted more as a consequence, than a cause, of the impact that rheumatic symptoms impose in the patients’ lifestyle, the impairment of their working potential, and the overall consequences in their life dynamics.

Our study had some limitations. We included only the Raramuri population settled in Chihuahua City, which possibly differs from the population residing in the more remote environments. As the sample did not include all the existing settlements, it may not fully represent the Raramuri population living in the city. However, it should also be noted that most of the individuals we sampled were born and raised in the Sierra and migrated to the city. The Sierra Tarahumara is the main area where the Raramuri live. It is a rural community characterized by forests and mountains, where the population is widely and sparsely distributed. The Raramuris in the rural environment walk longer distances (Raramuri means “those with light feet,” making reference to their ability to run very long distances) and carry out heavier work than the urban Raramuris, who have similar activities to the mestizo population, with most men working in low-wage construction jobs and women being housekeepers. Further investigation should include the rural Raramuri population.

Other limitations of our study were the scarcity of urban Raramuris and the non-availability of those in their indigenous lands; both situations conditioned a reduced sample. Limiting the questionnaire to urban Raramuris was the preferred strategy to fulfill the stages required by COPCORD. Furthermore, the Raramuri have a strict social structure and access to the settlements has to be granted by their settlement governor. For the largest settlement, such access was given but it was not unconditional. For this authorization, it was implicit that questionnaires had to be applied only during a certain time window of a few weeks, so it was not possible to return and include additional individuals. Fortunately, the acceptance to undertake the questionnaire was very good (93.9 %) probably due to the governor’s allowance and previous notification to the settlement and also to the association with the CET, whom they feel familiar with. The language barrier was also a limitation. Even if interviewers were capable of speaking the Raramuri dialect, the language has a restricted vocabulary. Thus, the meaning of several Spanish words, such as those related to pain, could not be adequately translated. This barrier could represent an underestimation of the pain intensity and its characteristics.

In conclusion, even though the Raramuri population had a lower prevalence of MSK pain than that reported for other indigenous groups, there was still a considerable proportion that suffered from non-traumatic MSK pain; possibly the lower prevalence of reported MSK pain is explained in part by a culturally induced stoicism caused by harsh life conditions over several generations. It is nevertheless evident that they are a vulnerable group for developing rheumatic diseases, which should be of concern to healthcare authorities who are too far to accomplish or fulfill their requirements. The most prevalent disease was OA and was concordant with the prevalence reported in the mestizo population in other regions in Mexico, and the prevalence of inflammatory rheumatic diseases is also similar. The epidemiologic data on the prevalence of MSK and rheumatic disorders permits the development of better healthcare and prevention programs for the Raramuri people.
